# Targeting PRMT1 Reduces Cancer Persistence and Tumor Relapse in *EGFR*- and *KRAS*-Mutant Lung Cancer

**DOI:** 10.1158/2767-9764.CRC-24-0389

**Published:** 2025-01-21

**Authors:** Xiaoxiao Sun, Karl Kumbier, Savitha Gayathri, Veronica Steri, Lani F. Wu, Steven J. Altschuler

**Affiliations:** 1 Department of Pharmaceutical Chemistry, University of California, San Francisco, San Francisco, California.; 2 Helen Diller Family Comprehensive Cancer Center, University of California, San Francisco, San Francisco, California.

## Abstract

Incomplete killing of cancer cells undermines oncogene-targeting therapies and drives disease relapse. Eliminating cancer cells that persist during treatment is crucial for improving treatment outcomes. Here, we discovered that a specific isoform of type I protein arginine methyltransferases (PRMT), namely, PRMT1, enables lung cancer cells with *EGFR* or *KRAS*^*G12C*^ driver mutations and high STAT1 activity to persist through targeted drug treatments. PRMT1 knockdown, combined with EGFR or KRAS^G12C^ inhibitors, decreased persistence and delayed cancer cell regrowth across cell line models and significantly prolonged tumor regression in xenograft models. In contrast, we found that knockdown of two other type I PRMT isoforms, PRMT4 and PRMT6, increased persistence. Finally, we found that targeting PRMT1 to reduce persistence is more effective in lung cancer models with intact versus deleted chromosome *5q31.1*, a region enriched with JAK-STAT pathway genes, suggesting a potential stratification criterion. Together, our study pinpoints the PRMT1 isoform as a critical vulnerability of cancer persistence in EGFR- or KRAS^G12C^-targeted therapies.

**Significance:**

Eliminating “persisters” before relapse is crucial for achieving durable treatment efficacy. This study provides a rationale for developing PRMT1-selective inhibitors to target cancer persisters and achieve more durable outcomes in oncogene-targeting therapies.

## Introduction

Targeted therapies that suppress oncogenic signaling are increasingly used to treat non–small cell lung cancer with driver mutations (currently in *EGFR*, *KRAS*, *ALK*, *RET*, *ROS1*, *NTRK*, *BRAF*, *ERBB2*, and *MET*; ref. 1). Despite initial response to these therapies, relapse is inevitable. For example, 80% of metastatic lung cancer patients with *EGFR*-activating mutations (*EGFR*^*mut*^) initially respond to osimertinib, a third-generation EGFR-targeting drug; yet, almost all experience tumor progression within 2 years, with a median duration of response of 17 months (2). In the case of the *KRAS*^*G12C*^ mutation in lung cancer, 30% of patients initially respond to sotorasib, a KRAS^G12C^-targeting drug, with a median response duration of only 9 months (3).

Relapse on targeted therapy can be caused by genetic mechanisms, notably the presence of tumor cells before treatment with pre-existing drug resistance mutations (4–6). However, relapse can also be caused by nongenetic survival mechanisms that allow cells to persist through treatment and ultimately evolve *de novo* resistance (7–9). An emerging strategy to enhance the efficacy and durability of current treatments is to target these therapy-persistent cancer cells [also referred to as “persisters” (10)] before relapse occurs. A number of persistence targets (including YAP/TEAD pathway, GPX4, APOBECs, and EIF4A) have emerged over the past decade (11–17). Yet, more targets and therapeutic approaches are needed to eliminate residual disease and prevent relapse (18–20).

Recently, we discovered a dependency of cancer persistence on type I PRMTs in lung cancer cells with high STAT1 signaling levels (21), which can arise from a number of factors, including intrinsic cancer cell signals, microenvironmental cytokines, and/or treatment-induced adaptation (21–28). Here, we investigated the antipersistence function of targeting type I PRMT isoforms in both *EGFR*^*mut*^ and *KRAS*^*G12C*^ lung cancer, using *in vitro* and *in vivo* models. Specifically, we investigated which specific PRMT isoform(s) are the targets, what additional oncogene-targeting therapies for lung cancer could benefit from combining with PRMT inhibition, whether the efficacy of PRMT inhibition observed in cell culture models translates to *in vivo* animal models, and what genetic alteration(s) can predict response to PRMT inhibition.

## Materials and Methods

### Cell lines and reagents

The origins and culture conditions for the cell lines are listed in Supplementary Table S6. Cells were tested by short tandem repeats fingerprinting to confirm their identities, and routinely screened for *Mycoplasma* and used within 10 to 15 passages after thawing. Other reagents used in the study are listed in Supplementary Table S7.

### Cell viability assay

For short-term persister emergence experiments, 1,000 to 5,000 cells per well were seeded in 96-well plates. Treatments started on the second-day postseeding and were replenished biweekly, continuing until the emergence of a persister cancer cell population was observed. The duration until emergence varied depending on the model, with a typical range of 1 to 2 weeks. Quantification of persister fitness at the conclusion of the treatment period was conducted using the CellTiter-Glo assay. For long-tern persister regrowth experiments, cells were seeded at a density of 10,000 cells per well in 96-well plates. Monitoring of cell fitness over extended periods was facilitated using the IncuCyte S3 Live Cell Analysis System (Sartorius; RRID:SCR_023147), which allowed for automated imaging. Daily acquisition of phase contrast images was conducted, capturing five fields per well using a 10× objective lens. The percentage of confluence for each well, at each time point, was quantified using the integrated analysis software provided with the IncuCyte system. In persister assays, unless otherwise indicated, cancer drug treatments were conducted at the following concentrations: erlotinib at 2.5 μmol/L, osimertinib at 100 nmol/L, and sotorasib at 1 μmol/L.

### RNA interference

For short-term knockdown experiments, a standardized transfection protocol was applied using SMARTpools siRNA (Dharmacon). Lyophilized oligonucleotides were reconstituted in siRNA buffer to 20 μmol/L, and aliquots were stored at −20°C. ON-TARGETplus SMARTpools consist of four different siRNAs targeting the same gene, and the nontargeting control pool was made up of four scramble siRNAs. A final concentration of 50-nmol/L siRNA was used for all experiments. siRNA was incubated with Lipofectamine 3000 transfection reagent (Invitrogen) for 15 minutes, and the siRNA–lipid complex was then added to the cells. Two days were allowed to facilitate gene knockdown before starting the downstream assessments either through persister assays or quantitative RT-PCR assays. For long-term knockdown experiments, a lentiviral transduction protocol was applied using SMARTvector Inducible Lentiviral shRNA (Dharmacon). Cells were seeded into six-well plates at a density of 1 × 10^5^ per well and then, 24 hours later, were infected with viral particles for 24 hours at 37°C with 8-μg/mL polybrene. Cells were selected in puromycin until a complete die-off was observed in the noninfected control cells. Knockdown efficiency was confirmed by Western blot. Reagents and materials used in RNA interference experiments are detailed in Supplementary Table S7.

### Quantitative RT-PCR for gene expression

RNA was extracted from cells using the RNeasy Plus Mini Kit (Qiagen). cDNA synthesis was conducted using the iScript Reverse Transcription Supermix (Bio-Rad), following the manufacturer’s protocols. Quantitative PCR was performed with the SsoAdvanced Universal SYBR Green Supermix (Bio-Rad). Relative mRNA expression levels were quantified against actin mRNA using the delta–delta threshold cycle (ΔΔCT) method. Details of the primers are available in Supplementary Table S8.

### Western blot

Denatured protein was resolved on Mini-PROTEAN gels (Bio-Rad) and transferred onto 0.45-μm polyvinylidene difluoride membranes. Membranes were incubated with Intercept blocking buffer (Li-Cor) for 30 minutes before incubation in primary antibodies at 4°C overnight on an orbital shaker. Membranes were washed 3 times for 5 minutes in TBS plus 0.1% Tween-20 before incubation in secondary antibodies for 1 hour at room temperature. Membranes were washed 3 times for 5 minutes again and scanned using Li-Cor Odyssey imager. Antibodies used in the study are listed in Supplementary Table S7.

### Animal studies

Xenograft studies were conducted at the Preclinical Therapeutics Core of the University of California San Francisco (UCSF). All animal handling, care, and treatment procedures were conducted in compliance with the UCSF Institutional Animal Care and Use Committee (IACUC)–approved animal protocol #AN194778. Female nude mice, ages 6 to 8 weeks old, were purchased from Envigo++++ (Athymic Nude-Foxn1nu, code 6904F) and housed with *ad libitum* food and water on a 12-hour light cycle at the UCSF Preclinical Therapeutics Core vivarium. Each mouse was subcutaneously inoculated on the right flank with 5 × 10^6^ cancer cells in 200-μL PBS supplemented with Matrigel (1:1). Tumor dimensions were measured with a digital caliper, and the tumor volume in mm^3^ was calculated using the formula: Volume = [(Width)^2^ × Length] × 0.52. Tumor sizes and body weights were measured twice weekly. The investigators performing measurements were not blinded. Animals were assigned into study groups via stratified randomization based on tumor volumes (*n* = 8–10 per group) when the average tumor size reached 500 mm^3^. Following stratified randomization, doxycycline was administered in drinking water at 1 mg/mL with 3% sucrose to induce the expression of PRMT1-targeting shRNA. After 3 days of doxycycline administration to ensure PRMT1 inhibition, daily cancer drug treatments were initiated via oral gavage. Osimertinib was dosed at 5 mg/kg with a formulation of 10% DMSO/40%PEG300/5% Tween-80; sotorasib was administered at 100 mg/kg with a formulation of 2% HPMC/1% Tween-80; and vehicle formulation served as the control. Cancer drug treatments were discontinued after tumors had shrunk and tumor volumes had stabilized, whereas doxycycline administration continued until the end of studies.

### *In silico* TCGA analysis

The Cancer Genome Atlas (RRID:SCR_003193) datasets for 506 lung adenocarcinoma (LUAD; ref. 29) and 478 lung squamous cell carcinoma (LUSC; ref. 30) patients were obtained from the Broad Firehose repository. Whole exome sequencing data for these patients was downloaded using R package maftools 2.18.0 (RRID:SCR_024519; ref. 31). Selected *EGFR* and *KRAS* mutations were determined based on indicators for targeted therapy and included *KRAS* p.G12C and EGFR p.E746_A750del, p.L858R, p.L747_T751del for *EGFR* mutations (32). Processed copy number variation (CNV) data for LUAD and LUSC patients were downloaded from the Broad Firebrowse repository (33). Somatic copy number alterations were called using Gistic2 (version 2.0.22, Firehose task version: 140; ref. 34) with the following parameters: *Amplification Threshold* = *0.1*, *Deletion Threshold* = *0.1*, *Cap Values* = *1.5*, *Broad Length Cutoff* = *0.7*, *Remove X-Chromosome* = *0*, *Confidence Level* = *0.99*, *Join Segment Size* = *4*, *Arm Level Peel Off* = *1*, *Maximum Sample Segments* = *2,000*, and *Gene GISTIC* = *1.* Boundaries for each somatic copy number alterations were based on wide peak limits.

### *In silico* PERC analysis

CNV data for 17 persister-derived erlotinib-resistant colonies (PERC) were taken from the original PERC publication (deposited at the Sequence Read Archive with accession number SRP068321; ref. 8). In short, sequences were aligned to human genome assembly hg19. Somatic CNVs were evaluated relative to PC9-1 (a clonal cell line derived from PC9 and used for generating PERCs) and called using MuTect (RRID:SCR_000559; ref. 35) with default parameters. We compared the distribution of persistence fitness scores between PERC groups with the *5q31.1* deletion present versus absent using two-sided Student *t* tests assuming unequal variance (Fig. 4E). We further evaluated the Kyoto Encyclopedia of Genes and Genomes pathway (RRID:SCR_018145) enrichment of genes within the *5q31.1* deletion region using a hypergeometric test (Supplementary Table S5). Finally, to assess the effect size of this deletion relative to other CNVs, we computed *t-*statistics (comparing PERC groups with an amplification or deletion present vs. absent) for amplifications or deletions present in at least three PERCs and no more than 14 PERCs (i.e., absent in at least three PERCs; Fig. 4F; Supplementary Table S4).

### Data and statistical analysis

For analysis of cell line data with endpoint readouts, pairwise comparisons between groups (e.g., experimental vs. control) were made using unpaired, two-sided Student *t* tests assuming unequal variance. For analysis of cell line confluence and xenograft tumor volume over time, comparisons between groups were based on confluence and relative tumor volume integrated over time (i.e., AUC). Statistical comparisons between groups (targeted drug + Dox vs. targeted drug alone) were made using one-sided Mann–Whitney tests, with the alternative hypothesis that targeted drug + Dox integrated confluence or tumor volume was less than targeted drug alone. Graphing of data was performed with GraphPad Prism software v.10.1.1 (RRID:SCR_002798) and R 4.3.3 (RRID:SCR_001905). Unless otherwise specified, data displayed are mean ± s.d. The meanings of symbols are as follows: not significant (ns), *P* > 0.05; *, *P* ≤ 0.05; **, *P* ≤ 0.01; ***, *P* ≤ 0.001; ****, *P* ≤ 0.0001.

### Data availability

The data generated in this study are available upon request from the corresponding authors. Whole exome sequencing data of PERCs used in this study are publicly accessible at the Sequence Read Archive under accession number SRP068321.

## Results

### PRMT1 is the key type I PRMT isoform mediating IFNγ/STAT1-promoted persistence

Pan-type I PRMT inhibitors can reduce persistence in cells with high STAT1 pathway activity (21). The type I PRMT family includes PRMT1, PRMT2, PRMT3, PRMT4, PRMT6, and PRMT8, which have been linked to various aspects of cancer development, including carcinogenesis, metastasis, and drug resistance (36–41). To search for isoforms responsible for STAT1-promoted persistence, we used various genetic and pharmacological approaches to perturb PRMT1, 2, 3, 4, and 6 (23); we excluded PRMT8 because of its brain-specific expression (37). We made use of the *EGFR*^*mut*^ NSCLC persister model PC9, which has tunable low or high STAT1 pathway activity in the absence or presence (respectively) of IFNγ stimulation and was used in the initial discovery of the type I PRMT vulnerability in persistent cancer cells (21).

**Figure 1:**
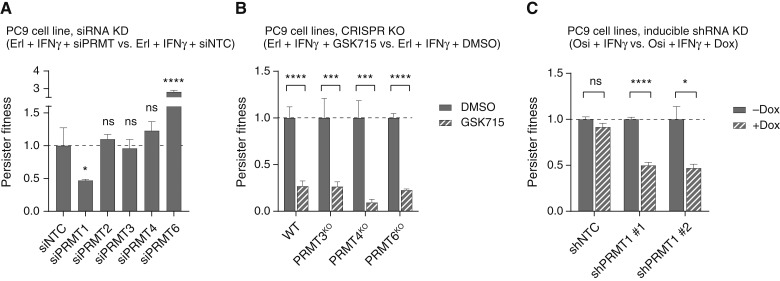
PRMT1 is the key type I PRMT isoform mediating IFNγ/STAT1-promoted persistence. **A,** Effects of type I PRMT knockdown (KD) via siRNA. PC9 cells were incubated with siRNA for 2 days to ensure gene inhibition. Subsequently, cells were treated with the EGFR drug erlotinib (Erl, 2.5 μmol/L) and IFNγ (5 ng/mL) for 6 days. Cell viability was measured, and persister fitness was normalized to nontargeting control siRNA (siNTC). **B,** Effects of pan-type I PRMT inhibitor GSK3368715 (GSK715) with PRMT knockout (KO) cells. PC9 cells with wild-type (WT) or deleted (KO) PRMTs were cotreated with erlotinib and GSK715 (0.2 μmol/L) in the presence of IFNγ for 6 days. Persister fitness was normalized to no GSK715 (DMSO) condition for each cell line. **C,** Effects of PRMT1 KD via inducible shRNA. Engineered PC9 cells expressing nontargeting control shRNA (shNTC) or PRMT1-targeting shRNA (siPRMT1 #1 and #2) were treated with the EGFR drug osimertinib (Osi, 100 nmol/L) in the presence of IFNγ for 6 days. Doxycycline (Dox, 100 ng/mL) was administered concurrently to induce PRMT1 knockdown. Persister fitness was normalized to the no Dox condition for each cell line. All data are reported as mean ± standard deviation, with three to five replicates per condition. Statistical comparisons between groups were based on unpaired, two-sided Student *t* tests. The significance of the results is indicated by symbols: not significant (ns), *P* > 0.05; *, *P* ≤ 0.05; ***, *P* ≤ 0.001; ****, *P* ≤ 0.0001.

First, using siRNA, we found that knockdown of PRMT1—but no other type I PRMT isoform—significantly reduced persistence under IFNγ stimulation (Fig. 1A; Supplementary Fig. S1A). This PRMT1 effect was specific to STAT1-promoted persistence under EGFR inhibition, as the reduction of persistence was not observed in the absence of IFNγ (Supplementary Fig. S1B) and cell fitness was not reduced in the absence of the EGFR drug (Supplementary Fig. S1C). Surprisingly, we observed that knockdown of PRMT6 significantly promotes persistence under IFNγ stimulation (Fig. 1A). These results suggest PRMT1 is the key isoform among type I PRMTs mediating IFNγ/STAT1 signal-promoted persistence.

Next, we investigated how the efficacy of pan-PRMT inhibition in persistence is affected by CRISPR knockout of distinct type I PRMT isoforms. We focused on PRMT1, 3, 4, and 6 and excluded PRMT2, as the siRNA knockdowns of PRMT2 did not show any persistence modulation activity (Fig. 1A; Supplementary Fig. S1B). Complete deletion of PRMT1 is lethal, consistent with the DepMap cell dependency data (https://depmap.org/portal; ref. 42). However, cell lines with PRMT3, PRMT4, or PRMT6 knockout were viable (Supplementary Fig. S1D). In these knockout cell lines, the pan-type I PRMT inhibitor GSK3368715 (43) did not lose its ability to significantly reduce persistence (Fig. 1B). These results supported the hypothesis that PRMT3, PRMT4, or PRMT6 are not the dominant isoforms responsible for the pan-PRMT inhibition’s ability to reduce persistence.

Finally, we used shRNA as an orthogonal method to knock down PRMT1. PC9 cells were engineered to express doxycycline-inducible shRNA targeting PRMT1 (Supplementary Fig. S1E). We found that doxycycline significantly reduced persistence levels upon treatment with the EGFR drug osimertinib and IFNγ (Fig. 1C). This effect was caused by doxycycline-induced PRMT1 knockdown, as no changes were observed by doxycycline in control cells expressing nontargeting control shRNA. Additionally, the shRNA knockdown of PRMT1 barely affected cell fitness in the absence of EGFR drug (Supplementary Fig. S1F). These results confirmed PRMT1 as the critical isoform mediating IFNγ/STAT1 signal-promoted persistence.

### PRMT1 knockdown decreases persistence in STAT1-high *EGFR*^*mut*^ and *KRAS*^*G12C*^ lung cancer cell lines

**Figure 2:**
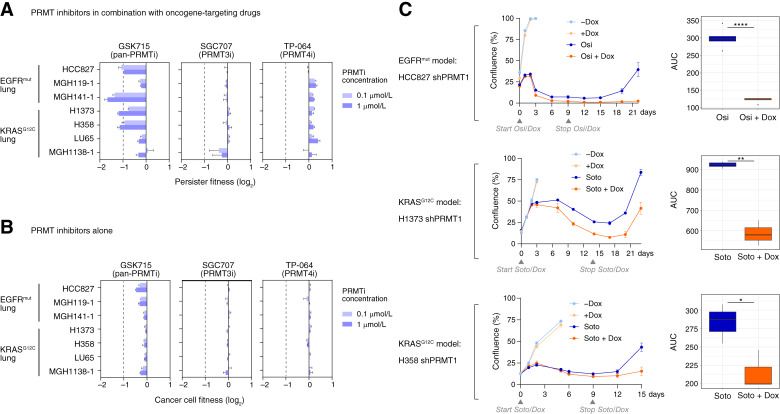
PRMT1 knockdown decreases persistence in STAT1-high *EGFR*^*mut*^ and *KRAS*^*G12C*^ lung cancer cell lines. **A** and **B,** Cells were treated with type I PRMT inhibitors (PRMTi) at 0.1 or 1 μmol/L, with (**A**) or without (**B**) targeted drugs. For EGFR^mut^ cells, the targeted drug was 100 nmol/L osimertinib, and for KRAS^G12C^ cells, the targeted drug was 1-μmol/L sotorasib. The pan-type I PRMT inhibitor GSK3368715 (GSK715), PRMT3-selective inhibitor SGC707, and PRMT4-selective inhibitor TP-064 were used. Persister fitness was calculated by normalizing cell viability after a six-day combination treatment to viability with targeted drug alone (**A**). General cancer cell fitness was calculated by normalizing cell viability after a three-day treatment with PRMTi alone to no PRMTi control (**B**). The graphs show the mean ± standard deviation (*n* = 3). **C,** Engineered lung cancer cells expressing PRMT1-targeting shRNA (shPRMT1) were treated with or without targeted drugs: 100 nmol/L osimertinib (Osi) for HCC827 shPRMT1 and 1-μmol/L sotorasib (Soto) for H1373 and H358 shPRMT1 cells. Doxycycline (Dox, 100 ng/mL) was administered concurrently with targeted drugs to ensure PRMT1 knockdown. Treatments lasted for at least 1 week before being discontinued to allow the regrowth of persister cells. The treatment schedules are indicated by gray triangles. Cells were continuously monitored using phase contrast live cell imaging. Cell confluence per well is reported as mean ± standard deviation, with three to five replicate wells per condition. Statistical comparisons between groups were based on the integrated confluence over time (area under curve; AUC), using one-sided Mann–Whitney tests. The significance of the results is indicated by symbols: *, *P* ≤ 0.05; **, *P* ≤ 0.01; ****, *P* ≤ 0.0001.

To evaluate more broadly the role of type I PRMT isoforms in persistence, we expanded our investigation to a collection of *EGFR*^*mut*^ and KRAS^G12C^ lung cancer cell lines. We found that these cell lines have relatively high intrinsic STAT1 pathway activity (compared with PC9 cells; Supplementary Fig. S2A) and that the pan-type I PRMT inhibitor (GSK3368715) decreased persistence (Fig. 2A). The relatively high STAT1 signaling levels in the cell lines allowed us to perform subsequent persister studies without IFNγ stimulation.

We first assessed the roles of PRMT3, PRMT4, and PRMT6. Little-to-no persistence-reducing activity was observed with the selective PRMT3 inhibitor SGC707 (44), whereas slight increases in persistence were seen with selective PRMT4 inhibitors TP-064 (45) and EZM2302 (Fig. 2A; Supplementary Fig. S2B; ref. 46). These inhibitors, tested at concentrations with reported cellular activities (Supplementary Table S1), showed minimal effects on general cell fitness (Fig. 2B; Supplementary Fig. S2B). Further, PRMT6 knockdown increased persistence across multiple cell lines with little impact on overall cell fitness (Supplementary Fig. S2C). These results indicated that inhibition of PRMT4 and PRMT6 would be counterproductive to eliminating persistence.

Next, we assessed the role of PRMT1. As there is currently no highly selective PRMT1 inhibitor, we engineered three of the STAT1-high cell lines (one *EGFR*^*mut*^ and two *KRAS*^*G12C*^) to express PRMT1-targeting shRNA (Supplementary Fig. S2D). These engineered cells were treated with either osimertinib for *EGFR* or sotorasib for *KRAS*^*G12C*^ mutations, alone or in combination with doxycycline-induced PRMT1 knockdown. The bulk population of the cells was sensitive to the oncogene-targeting drugs, with a fraction of cells persisting after week-long treatment. We withdrew the targeted drugs and monitored the regrowth of persistent cancer cells. In all three engineered cell models, PRMT1 knockdown alone induced little toxicity as it barely affected cell viability. Yet, the combination of PRMT1 knockdown with targeted drugs significantly reduced the regrowth of persistent cancer cells (Fig. 2C). These results supported the generality of PRMT1 as a vulnerability of persistence in STAT1-high lung cancers.

### PRMT1 knockdown enhances tumor regression in *EGFR*^*mut*^ and *KRAS*^*G12C*^ lung cancer xenograft models

**Figure 3:**
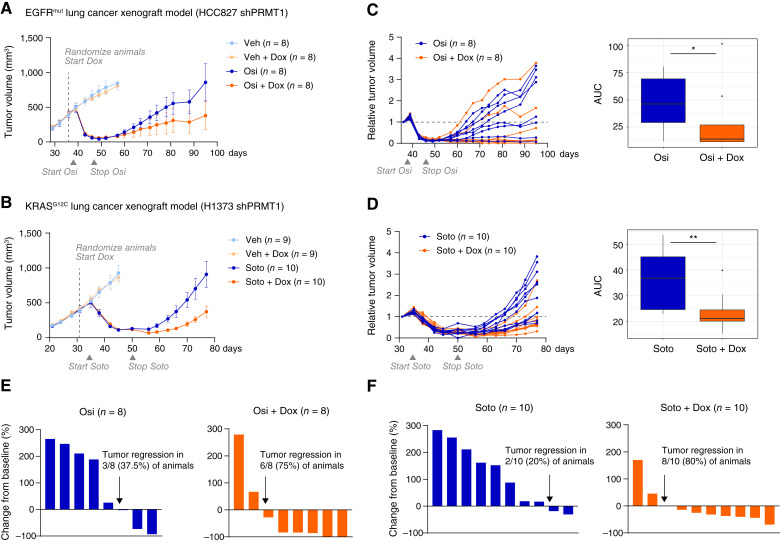
PRMT1 knockdown enhances tumor regression in *EGFR*^*mut*^ and *KRAS*^*G12C*^ lung cancer xenograft models. **A** and **B,***EGFR*^*mut*^ lung cancer cells HCC827 (**A**) and *KRAS*^*G12C*^lung cancer cells H1373 (**B**) were engineered to express PRMT1-targeting shRNA and subcutaneously injected into the flanks of nude mice. Once tumors reached an average volume of 500 mm^3^ (dashed lines), animals were randomly assigned to different groups (8–10 animals per group) and began receiving doxycycline (Dox, 1 mg/mL) in drinking water. After 3 days of Dox treatment to ensure PRMT1 knockdown, daily oral administration of either vehicle or targeted drugs began: 5 mg/kg osimertinib (Osi) for HCC827 and 100 mg/kg sotorasib (Soto) for H1373. Treatment with vehicle or targeted drugs was discontinued at the residual disease stage, whereas Dox administration continued until the end of the study. The dosing schedules of targeted drugs are marked with gray triangles. Tumor volumes were measured biweekly, and the data are presented as mean ± standard error. **C** and **D,** Tumor volumes of HCC827 (**C**) and H1373 (**D**) xenografts were normalized to baseline at randomization (dashed line) for each animal. Statistical comparisons between groups were based on the integrated tumor volume over time (area under curve; AUC), using one-sided Mann–Whitney tests. The significance of the results is indicated by symbols: *, *P* ≤ 0.05; **, *P* ≤ 0.01. **E** and **F,** Waterfall plots show the percentage change in tumor volume from baseline for individual animals at the conclusion of HCC827 (**E**) and H1373 (**F**) studies.

The persistence-reducing efficacy of PRMT1 reduction across cell lines motivated *in vivo* studies to investigate the potential of PRMT1 reduction to delay tumor relapse. We established targeted therapy-sensitive xenograft models using two lung cancer cell lines expressing PRMT1-targeting shRNA: one *EGFR*^*mut*^ model (HCC827) and one *KRAS*^*G12C*^ model (H1373). *In vivo* PRMT1 knockdown was confirmed upon doxycycline administration in both models (Supplementary Fig. S3A and S3B). Animals received oncogene-targeting drugs (osimertinib for *EGFR*^*mut*^ and sotorasib for *KRAS*^*G12C*^) alone or in combination with doxycycline-induced PRMT1 knockdown. As expected, tumors drastically regressed in response to the targeted drug treatments (Fig. 3A and B, dark vs. light blue curves), although residual tumors persisted in all animals (Supplementary Fig. S3C and S3D).

To accelerate tumor relapse, we withdrew the targeted drugs (as with the *in vitro* models; Fig. 2C) once tumor volumes stabilized and continued to monitor tumor progression. Both models showed that the combination with doxycycline-induced PRMT1 knockdown significantly reduced the tumor burden from the regression to progression stages (Fig. 3C and D). In the *EGFR*^*mut*^ model, 75% of the animals (six out of eight) receiving combination treatment remained tumor progression-free almost 3 months after the EGFR drug was stopped, including 25% (two animals) with complete regression (Fig. 3E). In contrast, only 37.5% of animals (three out of eight) in the osimertinib-only group were progression-free, with no complete regressions. Similarly, in the *KRAS*^*G12C*^ model, 80% of animals (eight out of 10) in the combination group remained progression-free compared with 20% (two out of 10) in the sotorasib-only group by the end of the study (Fig. 3F). Notably, PRMT1 knockdown alone did not affect tumor growth in the absence of targeted drugs (Fig. 3A and B). Furthermore, animal body weights remained unchanged with doxycycline, alone or in combination with the targeted drugs (Supplementary Fig. S3E and S3F). These results confirm that PRMT1 knockdown effectively enhances the efficacy and durability of EGFR and KRAS^G12C^ drugs in lung cancer xenograft models, demonstrating that the robust activity of the PRMT1-targeting strategy to eliminate persistence is translatable from *in vitro* to *in vivo*.

### Chromosome *5q31.1* deletion is negatively associated with the effectiveness of the PRMT1-targeting strategy for reducing persistence

**Figure 4:**
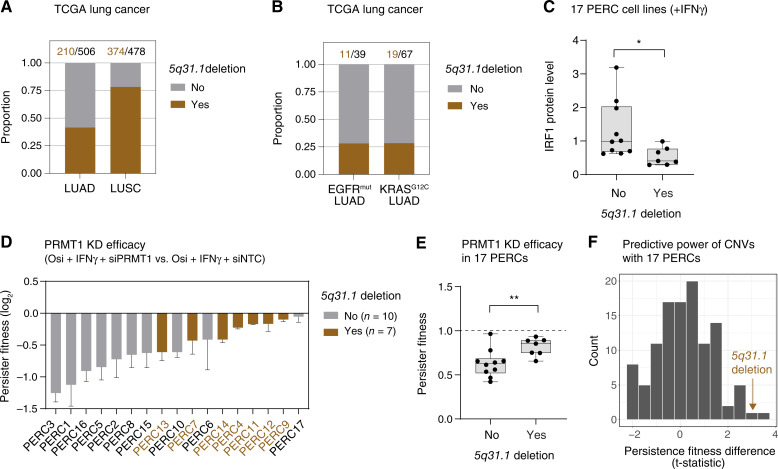
Chromosome *5q31.1* deletion is negatively associated with the effectiveness of the PRMT1-targeting strategy for reducing persistence. **A** and **B,** Chromosome *5q31.1* deletions in TCGA lung cancer cohorts. Proportions of LUAD and LUSC samples with (brown) or without (gray) deletion are shown in **A**. Proportions of samples with (brown) or without (gray) the deletion in LUAD harboring EGFR-activating mutations (*EGFR*^*mut*^) and *KRAS*^*G12C*^ mutations are shown in **B**. The numbers of samples with the deletion (brown) and the total cohort size (black) are labeled above the graphs. **C,** Box and whisker plots showing IRF1 protein levels in PERCs with or without the *5q31.1* deletion. Cells were treated with 50 ng/mL IFNγ for 2 hours. Protein levels were quantified from the Western blots in Supplementary Fig. S4C and normalized to the mean value across 17 PERCs. **D,** PRMT1 knockdown efficacy with PERCs. Cells were incubated with PRMT1-targeting siRNA (siPRMT1) for 2 days and then treated with 100 nmol/L osimertinib and 5 ng/mL IFNγ for 6 days. Persister fitness was calculated by normalizing cell viability to siNTC for each cell line. Data are shown as mean ± standard deviation (*n* = 3), ranked by the effect size of PRMT1 KD from large to small. Cell lines are color-labeled to indicate deleted (brown) or intact (gray) *5q31.1* region. **E,** Box and whisker plot showing PRMT1 knockdown efficacy between PERC groups with or without the *5q31.1* deletion. **F,** Distribution of *t*-statistics for PRMT1 knockdown efficacy between PERC groups with or without a copy number variation. Amplifications or deletions present in at least three PERC cell lines and no more than 14 PERC cell lines (i.e., absent in at least three PERC cell lines) were analyzed. Brown: the *5q31.1* deletion. Statistical comparison between groups was based on two-sided Student *t* tests assuming unequal variance. The significance of the results is indicated by symbols: *, *P* ≤ 0.05; **, *P* ≤ 0.01.

Given the effectiveness of targeting PRMT1 in reducing persistence in STAT1-high cancer cells, we reasoned that genetic alterations in the STAT1 pathway could provide clues for patient stratification. We searched for such genetic variations in lung cancer patients using The Cancer Genome Atlas data, focusing on a panel of 10 pathway-regulating genes: *IFNGR1*, *IFNGR2*, *JAK1*, *JAK2*, *PTPN2*, *STAT1*, *IRF1*, *PIAS1*, *SOCS1*, and *ISG15* (“Materials and Methods”). No prevalent point mutations in these genes were found in LUAD or LUSC (Supplementary Table S2). However, we identified two common trunk deletions in chromosome *1p36.13* (containing *ISG15*; Supplementary Fig. S4A) and chromosome *5q31.1* (containing *IRF1*; Fig. 4A). These deletions are prominent in *EGFR*^*mut*^ and *KRAS*^*G12C*^ LUAD, with one-third of *KRAS*^*G12C*^ LUAD carrying the *1p36.13* deletion (Supplementary Fig. S4B) and one-quarter of *EGFR*^*mut*^ or *KRAS*^*G12C*^ LUAD carrying the *5q31.1* deletion (Fig. 4B).

Studying the functional impact of large chromosomal deletions is challenging. Fortuitously, we discovered the presence of the *5q31.1* deletion in a subset of 17 previously established PERCs from the EGFR^mut^ PC9 cell line (Supplementary Table S3; “Materials and Methods”; ref. 8). Consistent with *IRF1*’s location within *5q31.1*, IFNγ stimulation revealed significantly higher IRF1 expression in the 10 PERCs without the *5q31.1* deletion compared with the seven with the deletion (Fig. 4C; Supplementary Fig. S4C).

The PERCs—evolved to resist the first-generation EGFR inhibitor erlotinib—show varying sensitivities to the third-generation EGFR inhibitor osimertinib. Using these models, we tested PRMT1 knockdown for its ability to reduce osimertinib-induced persistence under IFNγ stimulation. Notably, PRMT1 knockdown was more effective in PERCs without the *5q31.1* deletion than in those with the deletion (Fig. 4D and E). In fact, the *5q31.1* deletion ranked highest among all chromosomal deletions (and second highest among all CNVs) in its association with PRMT1 knockdown efficacy among the PERCs (Fig. 4F; Supplementary Table S4).

Various erlotinib resistance mechanisms are represented in the collection of PERCs (e.g., *EGFR*^*T790M*^, *NRAS* mutation, and *MET* or *RAF* amplification). Even among the seven *EGFR*^*T790M*^ PERCs, which are highly sensitive to osimertinib (Supplementary Fig. S4D), PRMT1 knockdown was more effective in reducing persistence in the four PERCs without the 5q31.1 deletion than in the three with it (Supplementary Fig. S4E). In contrast, no significant differences in persistence were observed for PRMT1 knockdown between *EGFR*^*T790M*^ PERCs and other PERCs (Supplementary Fig. S4F). Thus, *5q31.1* deletion seems to be more informative for predicting efficacy of targeting PRMT1 than an acquired resistance mechanism, such as *EGFR*^*T790M*^.

Finally, we investigated the *5q31.1* region for gene(s) contributing to resistance to PRMT1 targeting. Our analysis identified approximately 40 genes in the deleted region (Supplementary Table S4), with significant enrichment for JAK-STAT pathway genes including *IRF1*, which was used to identify this region in the first place (Supplementary Table S5). Interestingly, knockdown of *IRF1* alone did not significantly impact persistence with PRMT1 knockdown in three tested STAT-high lung cancer cell lines (Supplementary Fig. S4G). Further investigation is needed to identify genes that individually or in combination contribute to the differential responses to PRMT1 targeting. Nevertheless, our study reveals a negative association between the presence of *5q31.1* deletion and the efficacy of PRMT1 knockdown for reducing persistence.

## Discussion

Inhibiting PRMT family proteins has been proposed as a therapeutic strategy for cancer treatment. Multiple inhibitors of PRMT5, a type II PRMT protein, are currently in early clinical trials (47–51), and a pan-inhibitor of type I PRMTs entered phase 1 but was discontinued early because of a low chance of achieving a therapeutic window (52). To further develop PRMT-targeting modalities, especially for type I PRMTs, as cancer treatment, several critical questions need to be addressed.

First, which isoform(s) are critical to target, and which are dispensable or should be avoided to reduce toxicity? We identified their distinct functions in cancer persistence and found that PRMT1 is the essential isoform in the type I PRMT family to target for reducing STAT1-promoted persistence (Fig. 1A). Additionally, siRNA knockdown suggested that PRMT3, another type I PRMT isoform, contributes to persistence through yet unknown, non-STAT1 mechanisms (Supplementary Fig. S1B), Although we did not observe antipersistence efficacy with a PRMT3-selective inhibitor (SGC707; Fig. 2A), our results suggest that inhibiting PRMT3, at least, does not counteract the desired outcome of reducing persistence by targeting PRMT. In contrast, our data indicate that the other two type I PRMT proteins, PRMT4 and PRMT6, are counter-targets for persistent cancer cells: their knockdowns and/or pharmacological inhibitions promoted persistence across multiple *EGFR*^*mut*^ and *KRAS*^*G12C*^ lung cancer cell lines (Figs. 1A and 2A; Supplementary Figs. S1B, S2B, and S2C). Together, our data support developing inhibitors that are selective for PRMT1 and PRMT3, but not for PRMT4 and PRMT6, to eliminate cancer persistence.

Second, should PRMT inhibitors be used as monotherapy or in combination, and for which specific cancer types? By studying PRMT1 knockdown alone and in combination with oncogene-targeting drugs, we found that suppression of oncogenic signaling in lung cancer confers a collateral dependency on PRMT1 in persistent cancer cells. Data from orthogonal approaches (siRNA and shRNA knockdown) and various models (*EGFR*^*mut*^ and *KRAS*^*G12C*^ lung cancer; *in vitro* and *in vivo*) consistently show that knockdown of PRMT1 reduces persistence when combined with oncogene-targeting drugs but does not reduce general cell fitness when used alone. Given the many shared biological pathways of different oncogenic drivers, we speculate that a PRMT1-targeting strategy might have broader application in combination with other targeted therapies and in other cancer types (e.g., *ALK*-driven lung cancer, *KRAS*^*mut*^ colorectal cancer, or *HER2*-driven breast cancer). PRMT1 reduction demonstrates robust *in vivo* antipersistence activity and durable benefit. Future investigations are warranted to explore the use of combining current cancer-debulking therapies with novel persistence-eliminating PRMT1 inhibitors in more extensive disease settings beyond *EGFR*^*mut*^ and *KRAS*^*G12C*^ lung cancer.

Finally, what biomarker(s) can be used to predict the effectiveness of PRMT1-targeting strategies? First, an obvious candidate is PRMT1 expression itself. However, antipersistence effects of targeting PRMT1 were observed in cell lines with either high (e.g., PC9) or low (e.g., HCC827) PRMT1 levels (Figs. 2C and 3B; Supplementary Fig. S2A). Second, another possibility, guided by the literature, is *MTAP* status. Prior studies showed that type I PRMT and type II PRMT5 inhibitors are effective specifically in *MTAP*-deficient cells (43, 53); yet, our data in *MTAP*-intact cancer cells show that targeting PRMT1 reduces persistence (Figs. 2C, 3B, and E; Supplementary Fig. S2A). Thus, it is unclear whether *MTAP* status will serve as a biomarker in the PRMT1 persistence setting. Finally, we did discover that chromosome *5q31.1* deletion was negatively associated with the response to PRMT1 targeting. Intriguingly, the *5q31.1* region is enriched with JAK-STAT pathway genes (Supplementary Table S5). An open question for future investigation is which of the many genes in the 5q31.1 deleted region determines the effectiveness of targeting PRMT1.

Our study identified PRMT1 as the key type I PRMT isoform mediating persistence in STAT1-high lung cancer. We showed both *in vitro* and *in vivo* that PRMT1 knockdown enhanced the efficacy and durability of oncogene-targeting drugs in *EGFR*^*mut*^ and *KRAS*^*G12C*^ lung cancer models. We further identified that chromosomal deletion of 5q31.1 is a negative predictor of the effectiveness of the PRMT1-targeting strategy to eliminate persistence. Together, our results identify PRMT1, within the family of type I PRMTs, as the critical isoform to target for reducing cancer persistence in EGFR- or KRAS^G12C^-targeted therapies.

## Supplementary Material

Figure S1 Supplementary Figure S1 and legend

Figure S2 Supplementary Figure S2 and legend

Figure S3 Supplementary Figure S3 and legend

Figure S4 Supplementary Figure S4 and legend

Tables S1-S8 Supplementary Tables
