# Supplementary material for: Targeting PRMT1 Reduces Cancer Persistence and Tumor Relapse in EGFR- and KRAS-Mutant Lung Cancer
Source: Cancer Res Commun. 2025 Jan 21;5(1):119–27. doi: 10.1158/2767-9764.CRC-24-0389 (PMC11747858; doi:10.1158/2767-9764.CRC-24-0389)
Supplement: Figure S1 — Supplementary Figure S1 and legend [file crc-24-0389_figure_s1_suppsf1.docx]

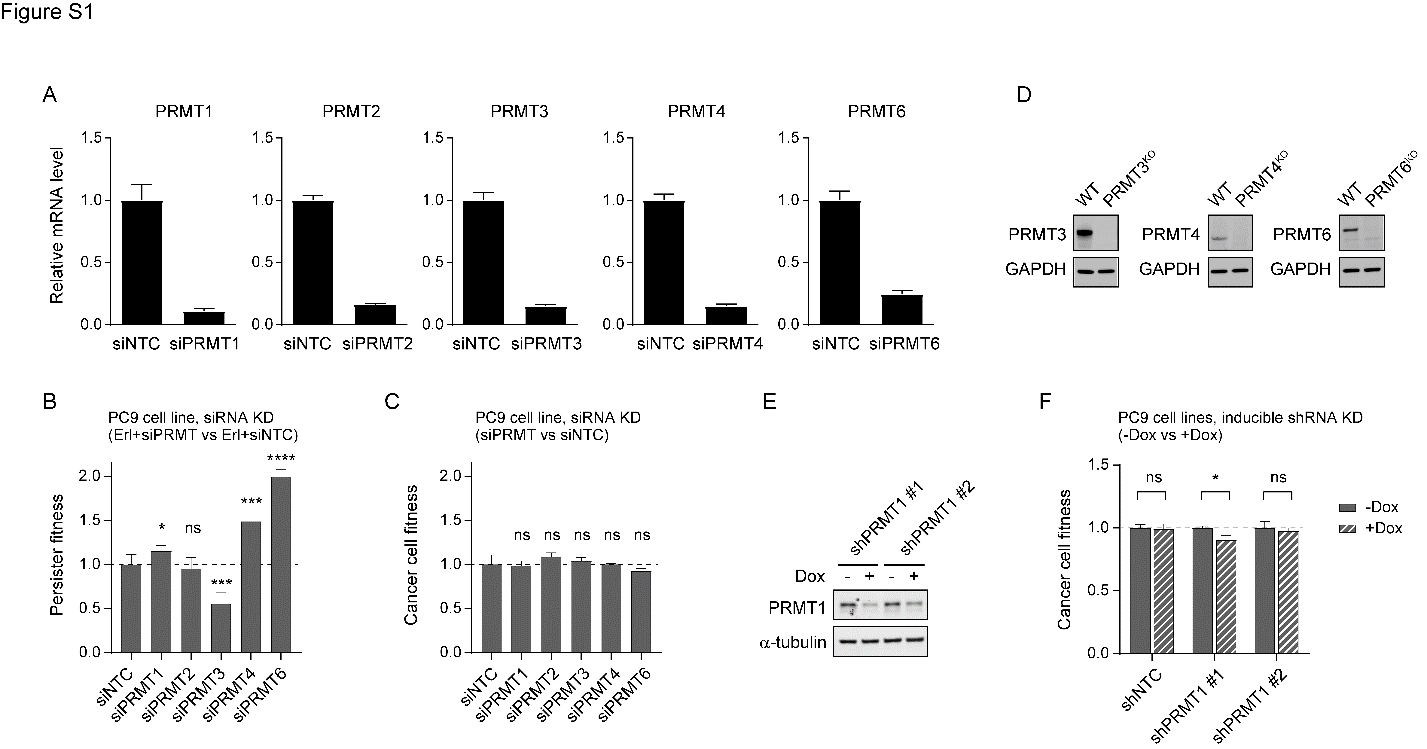


**Supplementary Figure S1. Validation of PRMT knockdown and knockout in PC9 cells.**

**A**. Verification of PRMT knockdown (KD) via siRNA. PC9 cells were incubated with siRNA targeting specific PRMTs (siPRMT) for 2 days. mRNA was subsequently extracted from the cells and analyzed via quantitative PCR. The relative mRNA levels with the indicated siPRMT were normalized to non-targeting control siRNA (siNTC). The graphs show the mean ± standard deviation (n=2). **B**-**C**. Effects of type I PRMT KD via siRNA. PC9 cells were incubated with siRNA for 2 days to ensure gene inhibition. Subsequently, cells were treated with the EGFR drug erlotinib (Erl, 2.5 μM) for 6 days (**B**) or cultured in pure media for additional 4 days (**C**) in the absence of IFNγ. Cell viability was measured and normalized to non-targeting control siRNA (siNTC). **D**. Verification of PRMT knockout (KO) via CRISPR. Cell lysates from PC9 cells with wild-type (WT) or deleted (KO) PRMTs were subjected to western blot analysis. The housekeeping protein GAPDH was used as a loading control. **E**. Verification of PRMT1 KD via inducible shRNA. Engineered PC9 cells expressing PRMT1-targeting shRNA (siPRMT1 #1 and #2) were treated with 100 ng/ml doxycycline (Dox) for 2 days to induce knockdown, followed by protein analysis using western blot. The housekeeping protein a-tubulin was used as a loading control. **F**. Effects of PRMT1 shRNA KD on general cancer cell fitness. Cells were cultured with or without Dox for 6 days. All cell fitness data are reported as mean ± standard deviation, with 3-6 replicates per condition. Statistical comparisons between groups were based on unpaired, two-sided Student’s t-tests. The significance of results is indicated by symbols: not significant (ns), *p* > 0.05; *, *p* ≤ 0.05; ***, *p* ≤ 0.001; ****, *p* ≤ 0.0001.
