# Supplementary material for: Targeting PRMT1 Reduces Cancer Persistence and Tumor Relapse in EGFR- and KRAS-Mutant Lung Cancer
Source: Cancer Res Commun. 2025 Jan 21;5(1):119–27. doi: 10.1158/2767-9764.CRC-24-0389 (PMC11747858; doi:10.1158/2767-9764.CRC-24-0389)
Supplement: Figure S2 — Supplementary Figure S2 and legend [file crc-24-0389_figure_s2_suppsf2.docx]

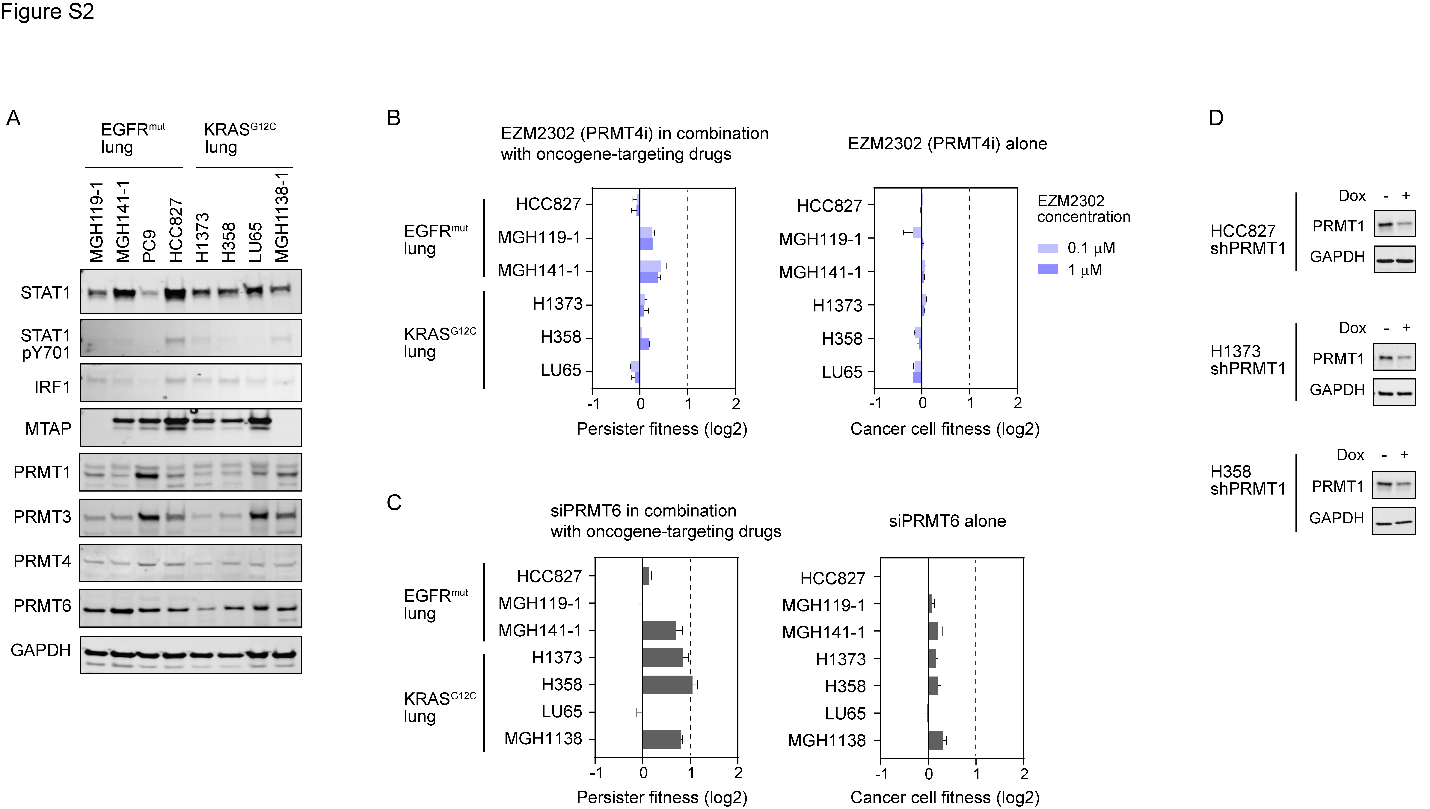


**Supplementary Figure S2. Targeting type I PRMTs in STAT1-high EGFR^mut^ and KRAS^G12C^ lung cancer cell lines.**

**A**. Cell lysates from 5 x 10^5 cells of each cell line were analyzed via western blot to assess the basal activity of the IFNγ/STAT1 signaling pathway and endogenous expression of type I PRMT proteins. PC9 cells were included as a reference due to their low basal activity of the STAT1 signaling. The housekeeping protein GAPDH was used as a loading control. The protein MTAP was included because its depletion has been reported as a biomarker for sensitivity to PRMT inhibition as monotherapy. **B**. Cells were treated with PRMT4 inhibitor (PRMT4i) EZM2302 at 0.1 μM or 1 μM, with (left) or without (right) targeted drugs (100 nM osimertinib for EGFR^mut^ cells, 1 μM sotorasib for KRAS^G12C^ cells). Persister fitness was calculated by normalizing cell viability after a 6-day combination treatment to viability with targeted drug alone. General cancer cell fitness was calculated by normalizing cell viability after a 3-day treatment with PRMTi alone to no PRMTi control. The graphs show the mean ± standard deviation (n=3). **C**. Effects of PRMT6 KD via siRNA. Cells were incubated with siRNA for 2 days to ensure gene inhibition. Subsequently, cells were treated with targeted drugs for 6 days (left) or cultured in pure media for additional 3days (right). Cell viability was measured and normalized to non-targeting control siRNA (siNTC). **D**. Verification of PRMT shRNA knockdown. Cells were treated with doxycycline (Dox, 100 ng/ml) for 2 days, and then cell lysates were subjected to western blot analysis. The housekeeping protein GAPDH was used as a loading control.
