# Supplementary material for: Targeting PRMT1 Reduces Cancer Persistence and Tumor Relapse in EGFR- and KRAS-Mutant Lung Cancer
Source: Cancer Res Commun. 2025 Jan 21;5(1):119–27. doi: 10.1158/2767-9764.CRC-24-0389 (PMC11747858; doi:10.1158/2767-9764.CRC-24-0389)
Supplement: Figure S3 — Supplementary Figure S3 and legend [file crc-24-0389_figure_s3_suppsf3.docx]

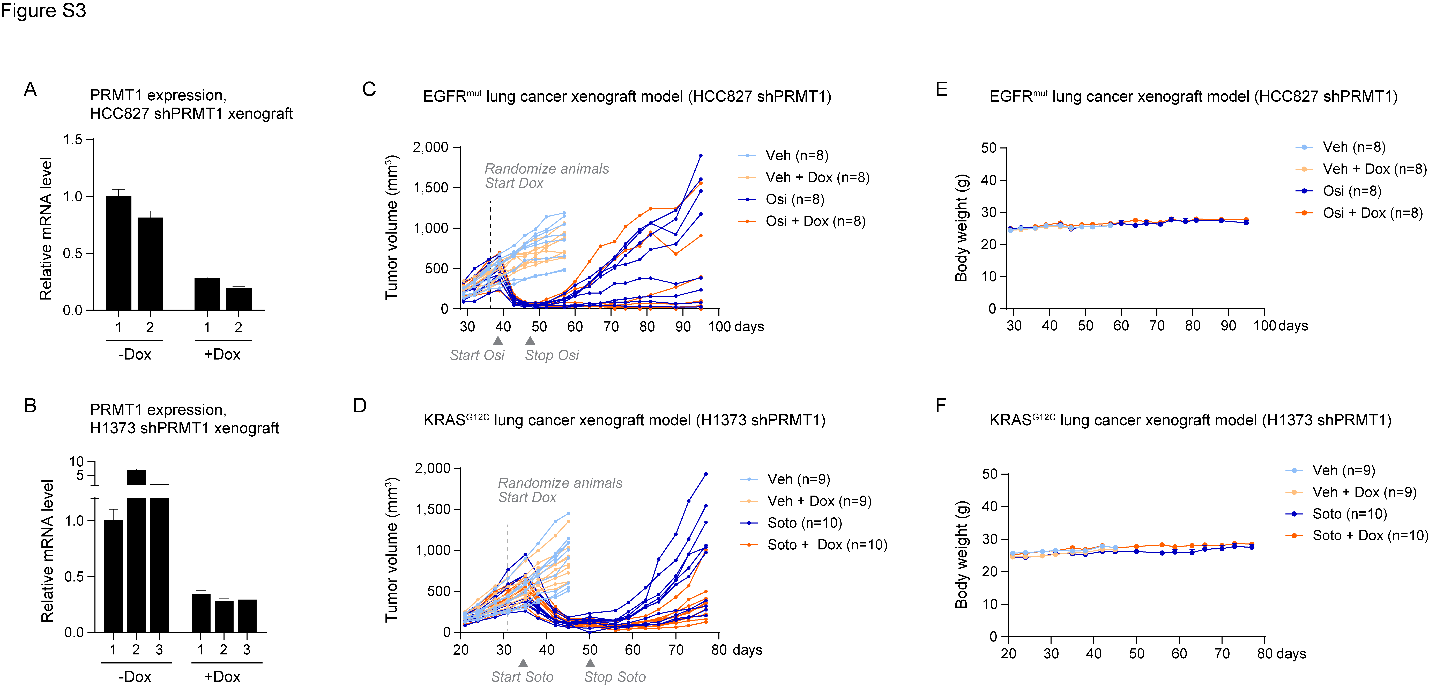


**Supplementary Figure S3. PRMT1 knockdown enhances tumor regression in EGFR^mut^ and KRAS^G12C^ lung cancer xenograft models.**

**A**-**B**. Validation of PRMT1 knockdown *in vivo*. Engineered lung cancer cells HCC827 (**A**) and H1373 (**B**) expressing PRMT1-targeting shRNA were subcutaneously injected into the flanks of nude mice. Three weeks post-injection, animals were administrated with or without 1 mg/ml doxycycline (Dox) in drinking water for 3 days. mRNA was then extracted from tumor tissues and analyzed via quantitative PCR to assess PRMT1 mRNA levels. The graphs show the mean ± standard deviation (2 to 3 animals per group, 3 technical replicates per animal). **C**-**D**. Tumor volumes of individual animals for HCC827 (**C**) and H1373 (**D**) xenograft studies. Cells were subcutaneously injected into the flanks of nude mice. Once tumors reached an average volume of 500 mm^3^ (dashed lines), animals were randomly assigned to different groups (8-10 animals per group) and began receiving Dox in drinking water. After 3 days of Dox treatment to ensure PRMT1 knockdown, daily oral administration of either vehicle or targeted drugs began: 5 mg/kg osimertinib (Osi) for HCC827, 100 mg/kg sotorasib (Soto) for H1373. Treatment with vehicle or targeted drugs was discontinued at the residual disease stage, while Dox administration continued until the end of the study. The dosing schedules of targeted drugs are marked with grey triangles. Tumor volumes were measured bi-weekly. **E**-**F**. Tolerability of PRMT1 knockdown in HCC827 (**E**) and H1373 (**F**) xenograft studies. Bi-weekly measurements of animal body weights are reported as mean ± standard error.
