# Supplementary material for: Targeting PRMT1 Reduces Cancer Persistence and Tumor Relapse in EGFR- and KRAS-Mutant Lung Cancer
Source: Cancer Res Commun. 2025 Jan 21;5(1):119–27. doi: 10.1158/2767-9764.CRC-24-0389 (PMC11747858; doi:10.1158/2767-9764.CRC-24-0389)
Supplement: Figure S4 — Supplementary Figure S4 and legend [file crc-24-0389_figure_s4_suppsf4.docx]

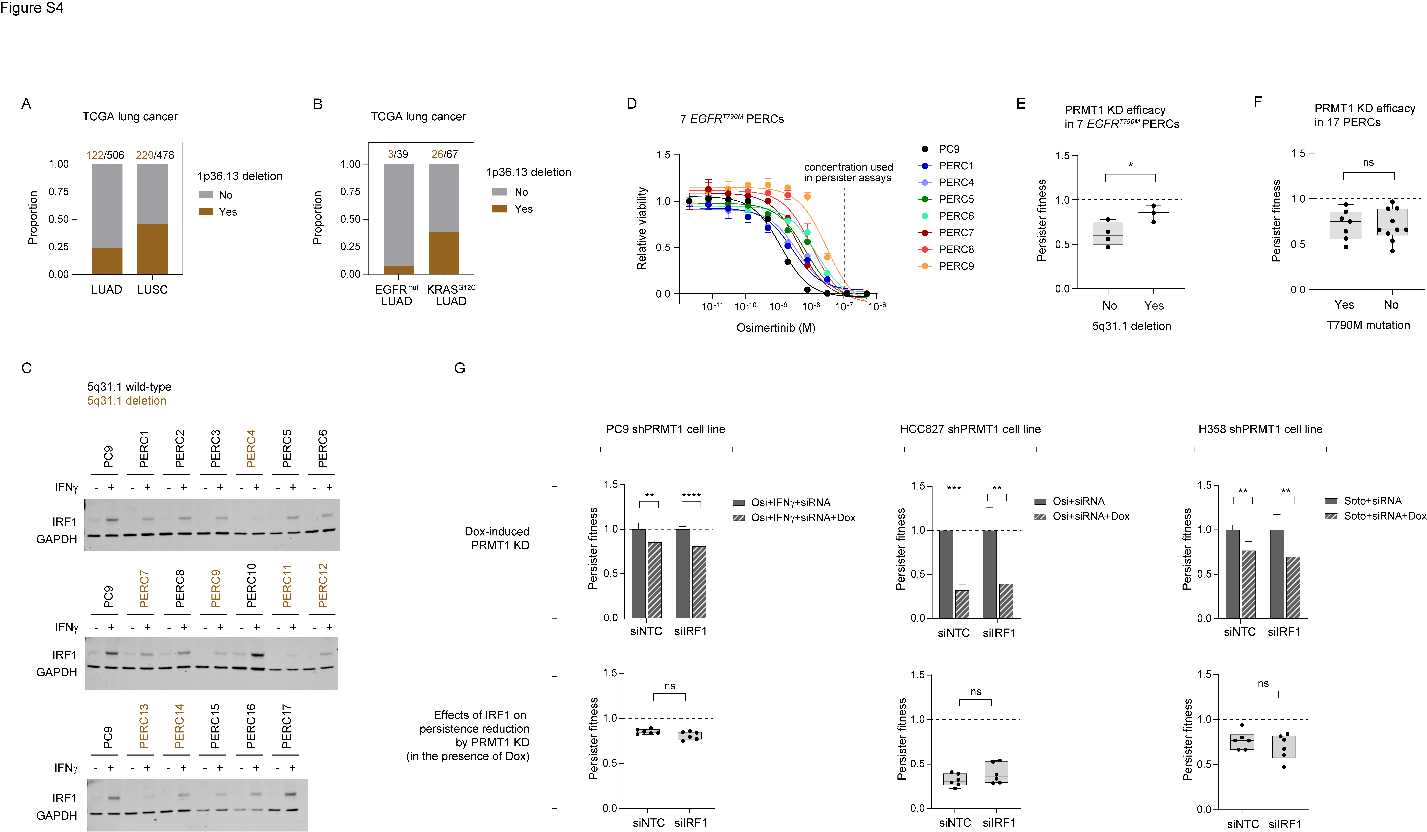


**Supplementary Figure S4. Genetic associations with the effectiveness of the PRMT1-targeting strategy for reducing persistence.**

**A**-**B.** Chromosome 1p36.13 deletions in TCGA lung cancer cohorts. Proportions of LUAD and LUSC samples with (brown) or without (grey) the deletion are shown in **A**. Proportions of samples with (brown) or without (grey) the deletion in LUAD harboring EGFR-activating mutations (EGFR^mut^) and KRAS^G12C^ mutations are shown in **B**. The numbers of samples with the deletion (brown) and the total cohort size (black) are labeled above the graphs. **C**. Cells were treated with or without 50 ng/ml IFNγ for two hours. PC9 was included in each blot as a reference to allow comparisons of PERCs across blots. The housekeeping protein GAPDH was used as a loading control. Cell lines are color-labeled to indicate deleted (brown) or intact (grey) 5q31.1 region. **D**. Osimertinib dose-response curves with PC9 and EGFR^T790M^ PERCs. Cells were treated for 4 days, and viabilities were measured using the CellTiter-Glo assay. Dashed line: osimertinib at 100 nM, the concentration used in persister assays. **E**. Box and whisker plot showing PRMT1 knockdown efficacy between EGFR^T790M^ PERC groups with or without the 5q31.1 deletion. **F**. Box and whisker plot showing PRMT1 knockdown efficacy between PERC groups with or without the EGFR^T790M^ mutation. **G**. Dox-inducible PRMT1 shRNA cells were incubated with non-targeting control siRNA (siNTC) or IRF1-targeting siRNA (siIRF1) for 2 days, and then treated with targeted drugs with or without 100 ng/ml doxycycline (Dox) for 6 days. Persistence models: Left, PC9 shPRMT1 cells with osimertinib treatment (Osi, 100 nM); Middle, HCC827 shPRMT1 cells with Osi treatment; Right, H358 shPRMT1 cells with sotorasib treatment (Soto, 1 μM). For each siRNA condition, persister fitness was normalized to the no Dox condition. For each persistence model: Top, bar plots showing mean ± standard deviation (n=6); Bottom, box and whisker plots showing persister fitness in the presence of Dox. Statistical comparisons between groups were based on unpaired, two-sided Student’s t-tests. The significance of results is indicated by symbols: not significant (ns), *p* > 0.05; *, *p* ≤ 0.05; **, *p* ≤ 0.01; ***, *p* ≤ 0.001; ****, *p* ≤ 0.0001.
